# Factors Associated with Human Papillomavirus Vaccination among Women in the United States

**DOI:** 10.20431/2456-0596.0301002

**Published:** 2018

**Authors:** Alexis Sims, Elaine Archie-Booker, Reinetta T. Waldrop, Mechelle Claridy, Gemechu Gerbi

**Affiliations:** 1 Master of Public Health Program, Morehouse School of Medicine, Atlanta, GA, USA; 2 Department of Community Health and Preventative Medicine, Morehouse School of Medicine, 720 Westview Drive SW, National Center for Primary Care (NCPC) Suite 346, Atlanta, GA 30310-1495, USA

**Keywords:** HPV, Vaccination, Cervical Cancer, Women

## Abstract

**Background:**

Human papillomavirus (HPV) infection is the most common sexually transmitted infection (STI) in the United States (US). HPV vaccines have the ability to prevent infection with HPV. The objective of this study was to assess the factors associated with HPV vaccination among women in the US.

**Methods:**

Data from the 2014 Behavioral Risk Factor Surveillance System were used to assess predictors of HPV vaccination. A multivariable logistic regression analysis was used to estimate adjusted odds ratios (AORs) and 95% confidence intervals (95% CIs). Analyses were conducted using SAS Version 9.4.

**Results:**

Factors that decreased the likelihood of receiving HPV vaccination included: being between the ages of 27–50 (AOR: 0.08; 95% CI: 0.06–0.11), having some college education, and residing in the South Black Belt States (AOR: 0.49; 95% CI: 0.31–0.78), Midwest (AOR: 0.63; 95% CI: 0.44–0.90), and the West (AOR: 0.37; 95% CI: 0.15–0.95). Factors that decreased the likelihood of receiving HPV vaccination to completion included: being Non-Hispanic Black (AOR: 0.26; 95% CI: 0.11–0.64), Hispanic (AOR: 0.26; 95% CI: 0.10–0.68), between the ages of 27–50 years (AOR: 0.46; 95% CI: 0.26–0.84), and residing in the Midwest (AOR: 0.36; 95% CI: 0.18–0.73) and South Remainder (non- Black Belt) states (AOR: 0.30; 95% CI: 0.09–0.93).

**Conclusion:**

Our results suggest that sociodemographic disparities still exist in more recent data underscoring the urgent need for additional efforts to increase HPV vaccination in populations that are least likely to receive the vaccination.

## 1. Introduction

Human Papillomavirus (HPV) is the most common sexually transmitted infection (STI) in the United States (U.S.); nearly 80 million people, about one in four, are currently infected in the U.S.^1^ HPV is so frequent that the majority of sexually active men and women are infected with the virus at some point during their lifetime. However, in most people, the body will typically clear the infection on its own and they do not get noticeably sick from the infection. Since Papanicolaou (Pap) smear screening was introduced in the 1950s, cervical cancer incidence has decreased over 80%.^2^ Even though this signifies an enormous public health achievement; it is possible for even greater positive effects on this disease with widespread uptake of the HPV vaccination.^2^

There are three Food and Drug Administration (FDA) approved HPV vaccines that have been on the market, Gardasil, Cervarix, and Gardasil 9.^3^ These three vaccines primarily prevent infections with HPV types 16 and 18, the two high-risk HPVs that cause about 70 % of cervical cancers.^4^ The HPV vaccine has traditionally been administered in three shots, however, as of October 2016, the Centers for Disease Control and Prevention (CDC) released new age-related recommendations for HPV vaccinations. The CDC recommends that adolescent girls and boys who are 11 to 12 years old receive two doses of the HPV vaccine rather than the previously recommended three doses.^5^ Girl and boys ages 15 and above will continue to receive three doses of the HPV vaccine.^5^ The Advisory Committee on Immunization Practices (ACIP) also approved HPV vaccination for all women from 11 to 26 years old, and all young men through age 21.^1^ Gardasil-9 will soon be the only HPV vaccine available in the US. The last doses of Cervarix expired at the end of November 2016 and the last does of Gardasil (quadrivalent HPV vaccine) will expire in May of 2017.

Since the HPV vaccine has been shown to be highly effective, even minor variances in access to vaccination could decrease the rate of disparities if vaccine acceptance is equitable throughout all populations.^6^ Even though HPV vaccines are readily available in the US and vaccine rates have been steadily increasing over the last few years, vaccination rates still remain low at about 63% for women.^6^ When examining the specific cancer-related consequences of HPV infection, cervical cancer is the most significant consequence, with over 500,000 new cases and 275,000 associated deaths globally in 2008.^6^ Low vaccination rates in women are strong indicators of increased rates of cervical cancer incidence across various populations. Another factor in low vaccine coverage is low rates of patient follow-up to complete the HPV three-dose series. Many women will begin the series and not finish. Only about 38% of eligible females get the complete, three-dose vaccination.^7^ This low rate of vaccination initiation and completion continue to contribute to the high prevalence of HPV infection

## 2. Methods

Data for this study were obtained from the 2014 Behavioral Risk Factors Surveillance System (BRFSS) (N=1,722). The BRFSS is the nation’s leading system of health-related telephone surveys that collects state data from a random sample of U.S. adults aged 18 years and older concerning their health-related risk behaviors, chronic health conditions, and usage of preventive services annually.^8^ BRFSS collects data from all 50 states along with the District of Columbia and three participating U.S. territories, Puerto Rico, Guam, and the US Virgin Islands.^8^ Each year BRFSS conducts over 400,000 adult interviews, which makes it the largest continuously conducted health survey system in the world. To account for differences in the probability of selection and non response, a clustering sample design was used to precisely derive U.S. and state-based population approximations. A primary set of questions are asked in the annual survey in all 50 states, the District of Columbia, Puerto Rico, Guam, and the U.S. Virgin Islands.

### 2.1. Measures

All measures in this study were based on the self-reported data obtained from the 2014 BRFSS.

### 2.2. Dependent Variables

#### 2.2.1. HPV Vaccination

HPV vaccination was defined by a respondent answering, “Yes” to the question: “Have you ever had an HPV vaccination?” Response categories were “Yes, “No”, “Don’t know/not sure”, or “Refused” responses. Only those records with “yes” or “no” responses were included in the analysis. Records with “unknown” or “refused” responses or missing data were excluded from the analysis to minimize underestimation.

#### 2.2.2. HPV Vaccination Completion

Completing HPV vaccine series was defined by a participant answering “All shots” to the question: How many HPV shots did you receive?” response categories were “1–2 shots,” “All shots,” “Don’t know/not sure”, or “Refused” responses. Only those with “1–2 shots” or “All shots” were included in the analysis. Records with “unknown” or “refused” responses or missing data were excluded from the analysis to minimize underestimation.

### 2.3. Independent Variables

#### 2.3.1. Socio-Demographic Measures

The independent variables obtained from the 2014 BRFSS survey participants include race or ethnicity, age, education, incomes, marital status, and region of residence. Records with “do not know/not sure”, “unknown” or “refused” responses or missing data were excluded from the analyses.

### 2.4. Analytic Plan

Univariate analyses were used to describe the frequency and proportion of select characteristics (age, race/ethnicity, level of education, level of income, region of residence, martial status). Bivariate analysis was performed to assess the initial assessment of factors independently associated with HPV vaccination and HPV vaccination completion. A multivariable logistic regression model was used to estimate adjusted odds ratios (ORs) and 95% confidence intervals (95% CIs) for factors associated with self-reported HPV vaccination and HPV vaccination completion. All predictors of HPV vaccination and HPV vaccination completion with a p-value of ≤ 0.05 in the bivariate variable analyses were entered into the multivariable logistic model. For the self-reported HPV vaccination model, these variables were race/ethnicity, age, level of education, level of income, marital status, and region of residence. For the self-reported HPV vaccination completion model, these variables were race/ethnicity, age, level of education, level of income, and region of residence. Bivariate and multivariable analyses excluded persons with responses that were missing or recorded as “don’t know/not sure” or “refused”. A 2-sided p-value<0.05 was considered to indicate statistical significance. Analyses were conducted using SAS version 9.4 (SAS Institute, Cary NC).

## 3. Results

The summary of the sociodemographic characteristics of the 1,722 women surveyed in the 2014 BRFSS is presented in Table 1. Of the 1,722 participants who responded to the question “Have you ever had an HPV vaccination?” 275 (16%) reported having had an HPV vaccination and 1,447 (84%) reported not having an HPV vaccination (Table 1). Of the 275 respondents who had an HPV vaccination, 72.4% were White, non-Hispanic; and 57.8% were between 18–26 years. Most of the respondents (40.7%) had some college education; had an annual household income of less than $24,999 (43.6%); were not currently married (78.2%); and were from the Midwest region of the United States (40.7%) (Table 1).

In multivariable logistic regression analysis, factors that decreased the likelihood of receiving HPV vaccination included: being between the ages of 27–50 (AOR: 0.08; 95% CI: 0.06–0.11), having some college education (AOR: 0.58; 95% CI: 0.38–0.89), and residing in the South Black Belt States (AOR: 0.49; 95% CI: 0.31–0.78), Midwest (AOR: 0.63; 95% CI: 0.44–0.90), and the West (AOR: 0.37; 95% CI: 0.15–0.95) (Table 2).

Factors that decreased the likelihood of receiving HPV vaccination to completion included: being Non-Hispanic Black (AOR: 0.26; 95% CI: 0.11–0.64), Hispanic (AOR: 0.26; 95% CI: 0.10–0.68), between the ages of 27–50 years (AOR: 0.46; 95% CI: 0.26–0.84), and residing in the Midwest (AOR: 0.36; 95% CI: 0.18–0.73) and South Remainder (non- Black Belt) states (AOR: 0.30; 95% CI: 0.09–0.93) (Table 3).

## 4. Discussion

The findings from this study suggest that socio-demographic and geographic disparities exist in HPV vaccination and HPV vaccination completion. In this study, being between the ages 27–50 decreased the likelihood of women receiving the HPV vaccination and the likelihood of receiving the vaccination to completion. The most possible explanation for this finding is the perception that the vaccination is not needed or as effective at an older age. Some older women may also perceive that HPV exposure has probably already occurred at some point in their lifetime. If women believe it is too late for them to benefit from HPV vaccination, lower vaccine uptake rates among women ages 26 and older will be observed. Another explanation is that many primary care physicians do not recommend or bring up getting vaccinated during appointments for their patients over 26.

For race and ethnicity, the factors that decreased the likelihood of receiving HPV vaccination to completion included being Non-Hispanic Black and Hispanic. A few possible explanations could be a racial gap in enthusiasm or trust in the vaccine^9^. This could explain why Non-Hispanic Whites were more likely to complete the vaccine series. Also, variances in economic assets including household income, access to community resources, health services, and related health information could all contribute to the disparities observed by race/ethnicity.^10^ Another explanation could be that lower HPV vaccination in these minority populations suggests that there may be some cultural differences that create barriers to vaccine initiation and completion.^11^ It is important that preventive information and HPV vaccine is targeted using culturally competent approaches to ensure these populations feel motivated to get vaccinated and also finish the full vaccine series.

In this study residing in the South Black Belt States, Midwest, and the West decreased the likelihood of receiving HPV vaccination. Also in this study, residing in the Midwest and South Remainder states decreased the likelihood of receiving HPV vaccination to completion. Rahman et al found that HPV vaccine uptake among young adult women was lowest in the south. Many southern states such as Georgia, Florida, Louisiana, Texas, and more have not chosen to expand Medicaid for their state, which may result in women from these states having fewer opportunities to get the health coverage they need for HPV vaccination.^12^ These non-Medicaid expanded states are the same states that need increased HPV vaccination uptake the most. There are pockets of certain communities residing in the South Remainder states and Midwest that have disproportionately lower HPV vaccination rates than other areas in the same states^12^. More targeted educational approaches must be used to focus on increasing vaccine uptake among these populations in the South Remainder states and Midwest. Also, state-level policies are likely to be a contribution to the geographic variances observed for HPV vaccination.^13^ This gives an indication of how large regional disparities in the U.S. for HPV vaccination could continue to grow if changes in policies are not implemented. Also In this study, having some college education was another factor that decreased the likelihood of receiving HPV vaccination. The possible explanations could include that lower education among women indicates less knowledge or less favorable attitudes about the benefits of HPV vaccination.^14^ Lower education and level of income have often been observed interchangeably as markers of lower HPV vaccine uptake.^14^

The strengths of this study are that BRFSS is a nationally representative dataset that is state based. This allows the study population to be representative of the U.S. population. This study also had some limitations. BRFSS excludes individuals without telephone service, those on military bases, and individuals in institutions. For this reason, generalizability to the entire U.S. population is limited. This study also relied on self-reported information, which may have introduced recall bias or reporting bias. Another limitation is that the age range 27–50 was included despite the recommended age cut-off of 26. Also, since the recommended age for vaccination is 11–12, this dataset also did not capture that population.

## Figures and Tables

**Table 1:** Number* and percentage of women who reported having had an HPV vaccination by select characteristics and health status: 2014 BRFSS, United States

Select Characteristics	Ever had a HPV Vaccination (N=1722)		
	Yes	No	p-value
	n (%)	n (%)	
All Females	275 (16%)	1447 (84%)	
Race/Ethnicity			<.0001
White, Non-Hispanic	199 (72.4%)	1074 (74.2%)	
Black, Non-Hispanic	32 (11.6%)	193 (13.4%)	
Asian, Non-Hispanic	3 (1.0%)	18 (1.2%)	
American Indian/Alaskan Native, Non-Hispanic	4 (1.5%)	20 (1.4%)	
Hispanic	26 (9.5%)	91 (6.3%)	
Other race, Non-Hispanic	11 (4.0%)	51 (3.5%)	
Total	275 (100%)	1447 (100%)	
Age Group			<.0001
18–26	159 (57.8%)	133 (9.2%)	
27–50	116 (42.2%)	1310 (90.8%)	
Total	275 (100%)	1443 (100%)	
Level of Education			0.0003
High School or less	74 (26.9%)	467 (32.3%)	
Some College	112 (40.7%)	493 (34.0%)	
College Graduate	89 (32.4%)	487 (33.7%)	
Total	275 (100%)	1447 (100%)	
Level of Income			0.0003
Less than $24,999	120 (43.6%)	623 (43.1%)	
$25,000 to $49,999	64 (23.3%)	321 (22.2%)	
$50,000 to $74,999	35 (12.7%)	188 (12.9%)	
$75,000 or more	56 (20.4%)	315 (21.8%)	
Total	275 (100%)	1447 (100%)	
Marital Status			<.0001
Currently Married	60 (21.8%)	694 (47.96%)	
Not Currently Married	215 (78.2%)	753 (52.04%)	
Total	275 (100%)	1447 (100%)	
Region of Residence			<.0001
South			
Black Belt States	44 (16.0%)	346 (23.9%)	
South Remainder	19 (6.9%)	60 (4.2%)	
Midwest	112 (40.7%)	576 (39.8%)	
Northeast	94 (34.2%)	377 (26.0%)	
West	6 (2.2%)	88 (6.1%)	
Total	275 (100%)	1447 (100%)	

**Note:**

* Frequencies may vary due to missing values

**Table 2:** Multivariable associations between women having had an HPV vaccination by select characteristics and health status: 2014 BRFSS, United States

Select Characteristics	Ever had a HPV Vaccination (N=275)	
	Adjusted OR	95% CI
Race/Ethnicity		
White, Non-Hispanic	Ref	
Black, Non-Hispanic	1.06	0.651–1.75
Asian, Non-Hispanic	0.39	0.097–1.62
American Indian/Alaskan Native, Non- Hispanic	1.24	0.362–4.51
Hispanic	1.07	0.616–1.88
Other race, Non-Hispanic	0.91	0.418–1.99
Age Group		
18–26	Ref	
27–50	0.08	0.05–0.11
Level of Education		
High School or less	0.89	0.61–1.29
Some College	0.58	0.38–0.89
College Graduate	Ref	
Level of Income		
Less than $24,999	0.91	0.53–1.56
$25,000 to $49,999	0.79	0.49–1.27
$50,000 to $74,999	0.74	0.47–1.18
$75,000 or more	Ref	0.74–1.93
Marital Status		
Currently Married	Ref	
Not Currently Married	1.88	1.28–2.74
Region of Residence		
South		
Black Belt States	0.48	0.30–0.78
South Remainder	1.255	0.65–2.40
Midwest	0.62	0.43–0.89
Northeast	Ref	
West	0.37	0.14–0.95

Note: OR=odds ratio; CI= confidence interval

**Table 3:** Multivariable associations between women having had a HPV vaccination completion by select characteristics and health status: 2014 BRFSS, United States

Select Characteristics	Received all three HPV Shots 178 (65%)	
	Adjusted OR	95% CI
Race/Ethnicity		
White, Non-Hispanic	Ref	
Black, Non-Hispanic	0.26	0.10–0.63
Asian, Non-Hispanic	0.08	0.00–1.17
American Indian/Alaskan Native, Non-	0.42	0.05–3.56
Hispanic		
Hispanic	0.26	0.10–0.66
Other race, Non-Hispanic	0.31	0.07–1.27
Age Group		
18–26	Ref	
27–50	0.46	0.25–0.83
Level of Education		
High School or less	0.97	0.43–2.20
Some College	1.30	0.64–2.66
College Graduate	Ref	
Level of Income		
Less than $24,999	0.92	0.39–2.13
$25,000 to $49,999	1.09	0.45–2.61
$50,000 to $74,999	0.96	0.33–2.70
$75,000 or more	Ref	
Region of Residence		
South		
Black Belt States	0.64	0.26–1.58
South Remainder	0.30	0.09–0.93
Midwest	0.35	0.17–0.73
Northeast	Ref	
West	0.23	0.04–1.39

**Note:** OR=odds ratio; CI= confidence interval.
